# Changing soil legacies to direct restoration of plant communities

**DOI:** 10.1093/aobpla/plx038

**Published:** 2017-08-23

**Authors:** E Pernilla Brinkman, Ciska E Raaijmakers, Wietse de Boer, Wim H van der Putten

**Affiliations:** 1 Department of Terrestrial Ecology, Netherlands Institute of Ecology (NIOO-KNAW), Droevendaalsesteeg 10, 6708 PB Wageningen, The Netherlands; 2 Department of Microbial Ecology, Netherlands Institute of Ecology (NIOO-KNAW), Droevendaalsesteeg 10, 6708 PB Wageningen, The Netherlands; 3 Department of Soil Quality, Wageningen University, PO Box 47, 6700 AA Wageningen, The Netherlands; 4 Laboratory of Nematology, Wageningen University, PO Box 8123, 6700 ES Wageningen, The Netherlands

**Keywords:** Fen meadow, grassland, nature restoration, nematode, plant community-soil feedback, soil biota, soil organisms

## Abstract

It is increasingly acknowledged that soil biota may influence interactions among plant species; however, little is known about how to change historical influences of previous land management on soil biota, the so-called ‘biotic soil legacy effect’. We used a two-phase plant community-soil feedback approach to study how plant species typical to original (i.e. undisturbed) and degraded fen meadows may influence effects of the soil community on *Carex* species that are dominant in fen meadows. In phase 1, soil from original, degraded, successfully and unsuccessfully restored fen meadows was conditioned by growing plants typical to original or to degraded fen meadows. In phase 2, interactions between *Carex* and neighbouring plant species were studied to quantify plant community-soil feedback effects in different neighbour plant mixtures. Soil conditioning with plants typical to original fen meadows resulted in significantly more *Carex* biomass than with plants typical to degraded fen meadows. These effects were strongest when the soil originated from unsuccessfully restored fen meadows. However, biomass of plants typical of degraded fen meadows was also higher in soil conditioned by typical fen meadow plants. We conclude that soil legacy effects of plants from degraded fen meadows can be altered by growing typical fen meadow plant species in that soil, as this enhances priority effects that favour growth of other typical fen meadow plants. As also plant species from degraded fen meadows benefitted from soil conditioning, further studies are needed to reveal if plant species can be chosen that change negative soil legacy effects for rare and endangered fen meadow plant species, but not for plant species that are typical to degraded fen meadows.

## Teaser

Soil life influences interactions among plant species. At the same time, soil life is the result of previous plant growth. These so-called ‘soil legacy effects’ may help to restore original vegetation in degraded fen meadows. We found that plants from degraded fen meadows cause negative soil legacy effects. These effects can be altered by growing typical fen meadow plant species in that soil. The challenge is to find plant species that change negative soil legacy effects such that rare and endangered fen meadow plant species will benefit, but not plant species of degraded fen meadows.

## Introduction

Changing abiotic soil conditions, such as reducing soil fertility and restoring original groundwater levels, has been shown insufficient to restore the characteristic plant community of species-rich grasslands (Marrs 1985; Grootjans *et al.* 2002). Rather, a combination of physical, chemical and biological measures may be required to facilitate the return of particular plant species that are typical to specific grassland communities (Heneghan *et al.* 2008). Here, we focus on biological measures, asking whether plants can be used to change the soil community to favour restoration of flora characteristic of undisturbed, species-rich wet grasslands (Wardle *et al.* 2004; Kardol and Wardle 2010). An understanding of how community interactions can contribute to ecosystem restoration has been identified as a major challenge for applied ecology (Eviner and Hawkes 2008; Harris 2009; Kardol and Wardle 2010).

Plants can induce soil legacy effects through changes in the composition of the associated soil community, which in turn influences the growth of the same or other plants (Bever 1994; Van der Putten *et al.* 2013). Belowground legacy effects of a preceding plant community can influence the outcome of competition between plant species, enhance plant species diversity by restricting potentially dominant plant species (De Deyn *et al.* 2004; Heinze *et al.* 2015; Jing *et al.* 2015) and may influence the success of restoration (Young *et al.* 2001; Grman and Suding 2010). Plant-soil feedback effects might be employed to restore the original plant communities of degraded ecosystems that have become dominated by early successional plant species (Kardol and Wardle 2010). Inoculation of a target soil community enhances the succession of grassland on former agricultural land (Middleton and Bever 2012) and directs plant community development into a specific vegetation type (Wubs *et al.* 2016). In contrast, without soil inoculation, it may take a long time before the soil community responds to a change in plant species composition (Elgersma *et al.* 2011). The aim of the present study was to assess if soil biota may be influenced to contribute to the restoration of degraded fen meadow associations (*Cirsio dissecti-Molinietum*) that have largely declined in Europe (Klimkowska *et al.* 2007).

Seed availability and establishment is crucial for successful restoration (Bakker and Berendse 1999). To date, most plant-soil feedback experiments have tested soil feedback effects on planted seedlings. Much less is known how soil legacies may affect seed survival and germination. Seed survival and germination can be affected by belowground saprophytes and pathogens (Gilbert 2002; Wagner and Mitschunas 2008). However, to our knowledge, it is not known if the soil organism effects on seed survival and germination are general and widespread, or whether they are specific and an existing plant community may modify them. In our experiment, we assessed plant-soil feedback effects on both seed germination and on biomass production of planted seedlings.

Plant-soil feedback experiments may focus on specific effects of certain plant species on others via a change in the soil community (Bever 1994), or may study effects caused by a group or community of plant species (e.g. Kardol *et al.* 2006). In this study, we focused on plant community-soil feedback effects to plants that are expected to become dominant in restored fen meadows. In a two-phase plant community-soil feedback experiment (Bever *et al.* 1997; Brinkman *et al.* 2010), we determined short-term effects of mixtures of plant species typical to original and to degraded fen meadows on soil nematodes, fungi and arbuscular mycorrhizal fungi. We assessed the subsequent influence of the soil community on seed germination, plant biomass production and the outcome of interspecific interactions between plants. We tested the hypotheses that: (i) plants typical to original fen meadows would influence the soil community to the benefit of *Carex* spp. that are common matrix species in original fen meadows, and (ii) this effect would be more pronounced in soil from original or successfully restored fen meadows than in soil from degraded or unsuccessfully restored fen meadows.

## Methods

Fen meadows are moist grasslands used for haymaking that occur on slightly acid to neutral sandy soils with a low availability of phosphorous (Schaminée *et al.* 1996; Grootjans *et al.* 2002). In winter the groundwater raises to the soil surface, while in summer the soils dry out superficially. Mowing and supply of base-rich groundwater are crucial to manage the vegetation composition. The vegetation of fen meadows is dominated by grasses (Graminae), sedges (Cyperaceae) and rushes (Juncaceae) that typically do not grow taller than 20 cm. Drainage, acidification and eutrophication are common causes of fen meadow degradation, initiating disappearance of the original plant community (Klimkowska *et al.* 2010). Degraded fen meadows are typically dominated by native, tall, fast-growing and competitive grasses or sedges (Joyce 2014). Measures that are used to restore fen meadows include restoration of hydrological conditions, removal of the nutrient-rich topsoil and introducing seeds by spreading hay collected from original fen meadows (Klimkowska *et al.* 2010). Restoration measures are considered successful when the intended fen meadow vegetation is re-established (Grootjans *et al.* 2002; Klimkowska *et al.* 2007). Restoration efforts are considered unsuccessful when characteristic fen meadow plants fail to return. *Juncus effusus* (Soft rush) dominates unsuccessfully restored fen meadows on former agricultural land (Smolders *et al.* 2008).

We classified fen meadows based on the presence (+) or absence (−) of a typical fen meadow vegetation according to Schaminée et al. (1996), and the history of the area: original (O) or restoration measures taken (R) (see Kemmers *et al.* 2013). We compared four meadow types:

- O+: original, undisturbed fen meadow with characteristic vegetation;
- O−: original fen meadow that has degraded and from where the characteristic vegetation has disappeared due to drainage and/or fertilization;
- R+: successfully restored fen meadow as based on the return of characteristic fen meadow vegetation;
- R−: unsuccessfully restored fen meadow as based on failure of return of characteristic vegetation.

**Figure 1.**
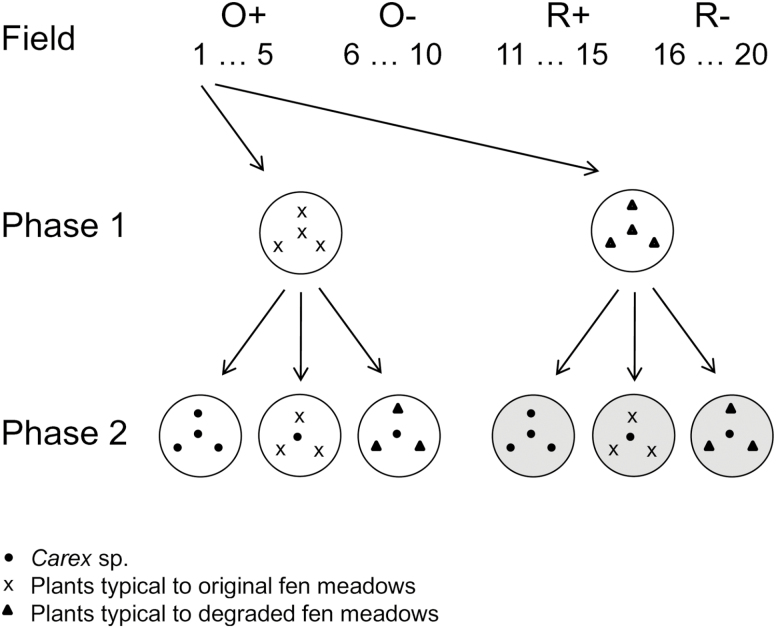
Experimental design. Soil was sampled from five fields each of four field types: original with characteristic fen meadow vegetation (O+; fields 1–5), original, but with degraded vegetation (O−; fields 6–10), successfully restored to characteristic fen meadow vegetation (R+; fields 11–15), unsuccessfully restored (R−; fields 16–20). In the first phase of the experiment, all the soils were conditioned with a mixture of plants characteristic to either original or to degraded fen meadows; as an example, the treatment of soil from field 1 is shown. In the second phase of the experiment, *Carex flacca*, *Carex hostiana* and *Carex panicea* were each grown surrounded by conspecifics or by plants characteristic to either original or to degraded fen meadows (see text and **Supporting Information—Table S2** for a list of plant species).

There were five replicate fields of each type of fen meadow across the mid and northeast of the Netherlands **[see Supporting Information—Table S1]**. From these (4 × 5 =) 20 fields, we collected soil for a two-phase plant community-soil feedback experiment (Bever *et al.* 1997; Kardol *et al.* 2006). In the first, soil conditioning phase of the experiment, mixtures of different plant species were grown in soils from each fen meadow type, thus possibly stimulating the development of specific soil communities. In the second phase, the response of seedlings of fen meadow *Carex* spp. to conditioned soils was tested for different mixtures of plant species (Fig. 1).

### Soil origin and plants

#### Soil.

In January and February 2008, we collected soil samples from all 20 fields. About 15–20 soil cores of 2.5 cm diameter, 0–20 cm depth were collected according to a stratified, W-shaped pattern and stored at 4 °C for a maximum of 1 week until further processing. The high soil moisture prohibited gentle separation of soil and roots, which is important to protect soil organisms like nematodes. Instead, the soil samples, including the roots, were cut into 2–3 cm pieces and then homogenized, removing stones when encountered. Soil samples were taken for analysis of abiotic characteristics and nematodes **[see Supporting Information—Appendix S1]**. The remaining soil was stored for 2–3 months at 4 °C, coinciding with winter inactivity of the soil biota, until the start of phase 1 of the experiment.

#### Plant species.

We used mixtures of plant species typical to original and to degraded fen meadows; in the first phase of the experiment to condition the soil and in the second phase as competitive neighbourhoods around the phytometers (Fig. 1). As a mixture of species characteristic of original fen meadows, we used *Festuca rubra* ssp. *commutata* (Chewing’s fescue), *Luzula multiflora* (Heath wood-rush), *Succisa pratensis* (Devil’s bit scabious) and, though only in the first phase of the experiment, *Juncus conglomeratus* (Compact rush) (Schaminée *et al.* 1996). As a mixture of species characteristic of degraded fen meadows, we used *Filipendula ulmaria* (Meadowsweet), *J. effusus* and *Lysimachia vulgaris* (Yellow loosestrife) (R. H. Kemmers, pers. comm.). The species that are characteristic to degraded fen meadows normally occur at lower soil pH and higher soil fertility than the original fen meadow species, as indicated by their Ellenberg values **[see Supporting Information—Table S2]**. We used three sedges as phytometers in phase 2 of the experiment. The three sedges all are dominant in fen meadows, but occur in other plant associations as well **[see Supporting Information—Table S2]**. The sedges increase in their degree of association to fen meadows in the following order: *Carex flacca* (Glaucous sedge), *C. panicea* (Carnation sedge) and *C. hostiana* (Tawny sedge) (Schaminée *et al.* 1996).

#### Seed origin and germination conditions.

Seeds of *C. hostiana* and *C. panicea* were collected from a fen meadow close to Wageningen, the Netherlands (52.01°N, 5.60°E). The seeds were air-dried at room temperature. Seeds of *F. rubra* were obtained from Medigran (Hoorn, The Netherlands) and seeds of the other plant species from B&T World Seeds (Aigues-Vives, France). The seeds were surface-sterilized in a 2 % hypochlorite solution for 1–2 min, and then rinsed with demineralized water. The seeds were sown on sterilized glass beads, moistened with demineralized water and stratified for 2 weeks at 4 °C with a 16/8 h light/dark regime. After stratification, the seeds were germinated at 20–30 °C with a 16/8 h light/dark regime. Seeds of *J. effusus* were germinated at the same greenhouse conditions as were used for the main experiment.

### Feedback experiment: phase 1

The soil collected from each of the five replicate fields of the four field types was mixed with sterilized background soil in a proportion of 1:6, based on dry mass, to avoid confounding effects of differences in soil physics and chemistry among fields. The background soil was collected from a former agricultural field where in the previous year the nutrient-rich topsoil had been removed, sieved with a 1 cm mesh size sieve, homogenized and sterilized by γ-irradiation (≥25 kGy). This dosage is known to effectively kill all soil biota (McNamara *et al.* 2003). Pots with a volume of 4 L (21 cm top diameter, 18 cm tall) were filled with the moist soil mixture (equivalent of 5.70 kg dry mass per pot). To obtain sufficient amounts of conditioned soil, for each treatment replicate in phase 1 we filled three pots with soil, whereas after conditioning the soil of the three pots was mixed and used as a substrate in phase 2. This procedure enabled us to condition ample amounts of soil while keeping the true replicates (the five fields per field type) independent. The pots were planted with either seedlings typical to original or to degraded fen meadows. In spite of seedling limitation we ensured that at least one seedling of each plant species was planted in one of the three pots, making sure that all the replicates 1 were planted with the same number of individuals of a species, as well as the replicates 2 and 3, creating similar plant configurations among the replicate pots of the fields. This resulted in 40 soil treatments (2 conditioning plant mixtures × 4 field types × 5 replicate fields).

In order to mimic moist fen meadow conditions, the pots were placed in individual containers with a water level set to 12 cm below the soil surface. Three times per week, the water table was reset using demineralized water and weed seedlings were removed. The pots were placed randomly on trolleys, which were rotated once every week to minimize effects of differences in microclimate in the greenhouse. The plants were grown for 20 weeks under controlled greenhouse conditions at 21/16 °C with a 16/8 h light regime. If needed, the plants were provided with extra light to ensure a minimum photosynthetic photon flux rate of 200 µmol m^−2^ s^−1^.

At harvest, the plants were clipped at ground level. The clipped shoot material was separated by species, dried at 70 °C for at least 48 h and weighed. Soil samples were taken for analysis of nematodes, ergosterol (an indicator of fungal biomass other than arbuscular mycorrhizal fungi (AMF)), neutral lipid fatty acids (NLFA; a measure of AMF) and abiotic soil characteristics **[see Supporting Information—Appendix S1]**. The remaining roots were clipped in 1–2 cm pieces, mixed with the remaining root zone and bulk soil and stored at 4 °C for 5–6 weeks until the start of phase 2 of the experiment.

### Feedback experiment: phase 2

Pots with a volume of 0.9 L (13 cm top diameter, 11 cm tall) were filled with an equivalent of 1.16 kg dry soil from phase 1 and placed in individual dishes. After that, the water level was set to 9 cm below the soil surface to simulate moist fen meadow conditions. Four seedlings were planted in each pot: one *Carex* sp. in the middle as a phytometer, surrounded by neighbourhood plantings of (i) three seedlings of the same *Carex* species, (ii) one seedling each of three other plant species that are typical to original fen meadows or (iii) one seedling each of three other plant species that are typical to degraded fen meadows (see description of **Plant species** and **Supporting Information—Table S2**). This resulted in 360 pots (4 field types × 5 replicate fields × 2 conditioning treatments × 3 *Carex* species × 3 neighbourhood plantings). The plants were allowed to grow for 14 weeks under the same greenhouse conditions as in phase 1. At harvest, the soil was washed from the roots. The roots of the different plant species in each pot were carefully separated. Shoot and roots were separated, dried at 70 °C for at least 48 h and weighed.

### Germination experiment

The effect of conditioning was tested on seed germination of *C. hostiana*, which is the most typical to fen meadows of the three *Carex* species. Square containers of 10 cm length × 10 cm width × 6 cm height were filled with an equivalent of about 0.38 kg dry soil. Only soils were used that originated from O+ and O− fields and that were conditioned with either plants typical of original or of degraded fen meadows, resulting in 20 containers (2 field types × 5 replicate fields per field type × 2 conditioning treatments). In every container, 49 seeds of *C. hostiana* were sown in a grid pattern. The containers were closed with perforated translucent lids and stratified at 4 °C for 2 weeks, after which they were transferred to the same greenhouse conditions as in phase 2 of the feedback experiment. The emerged seedlings were counted every 2–3 days during a period of 52 days.

### Data analysis

Conditioning soil from each field with two plant mixtures possibly diverged each soil community into two directions. As these treatments started from the same soil sample, they were treated as paired observations.

#### Soil characteristics.

Differences in abiotic characteristics and nematode communities among the field-collected soils from the four types of fen meadows were analysed, as well as effects of conditioning on abiotic soil characteristics, nematodes, ergosterol and NLFA **[see Supporting Information—Appendix S1]**.

#### Plant community conditioning effects.

At the end of the conditioning phase, the effects of field type and plant mixture on phase 1 plant shoot dry biomass were tested with two-way ANOVA using Statistica. At the end of phase 2, the effect of soil conditioning on the plants was calculated as a pairwise ratio for each soil origin (field) and neighbour plant mixture separately: ln[(biomass of *Carex* when grown in soil conditioned with plants characteristic of original fen meadows)/(biomass of *Carex* when grown in soil conditioned with plants characteristic of degraded fen meadows)]. Treatment means were based on mean values of the three *Carex* species. The low number of replicates did not allow for testing differences among the different neighbour plant mixtures. Therefore, within each neighbourhood treatment, differences among field types were tested with one-way ANOVA and Tukey HSD (*P* < 0.05). The difference of the conditioning effect from zero, representing no difference between the two conditioning treatments, was tested with *t*-tests. False discovery rates were controlled with the sharpened method of Benjamini and Hochberg (2000). This method reduces the chance of making type I errors, while having more power than classical Bonferroni-type control of family-wise error rate (Verhoeven *et al.*, 2005). Further, the correlation between shoot dry biomass in phase 1 and total dry biomass in phase 2 was tested with Pearson product-moment correlation using Statistica.

#### Germination experiment.

For each field, pairwise differences were calculated between germination of *C. hostiana* seeds in soil conditioned with plants typical of original and of degraded fen meadows. At each time point, *t*-tests were used (Statistica 10) to test if the difference in germination differed from zero. The effect of field type (O+ and O−) and time was tested with repeated measures ANOVA and Tukey HSD (*P* < 0.05).

## Results

### Soil characteristics

Abiotic soil properties did not significantly differ among, but showed high variance within the four field types **[see Supporting Information—Table S1]**. Soil conditioning by plants from original or degraded fen meadows neither significantly influenced the concentration of ergosterol, an indicator of fungal biomass, in the root zone soil, nor the concentration of NH_4_^+^ + NO_3_^−^ and Olsen-P **[see Supporting Information—Table S3]**. However, the concentration of the mycorrhiza-specific neutral lipid fatty acids (NLFA 16:1ω5) was significantly higher in soils that were conditioned with plants from original than from degraded fen meadows **[see Supporting Information—Table S3]**. These effects of soil conditioning were independent of field type.

PCA of the nematode community in the field-collected soil did not show a comprehensible pattern across the different field types **[see Supporting Information—Fig. S1]**. However, nematode density in the field-collected soil was 40-fold higher than in the conditioned soil. There were no significant differences between the two conditioning treatments in the density of endoparasites, ectoparasites, root hair feeders, bacterial and fungal feeders, and omnivores and predators **[see Supporting Information—Table S3]**.

### Plant community conditioning effects

Field source of inoculum did not significantly affect phase 1 shoot biomass (*F*_3,16_ = 0.46, *P* = 0.71). The shoot biomass of the plant mixture typical to degraded fen meadows (average 17.1 g and 95 % confidence interval 14.6–19.6 g) was greater than that of the plant mixture typical to original fen meadows (6.5 g (5.5–7.4 g); *F*_1,16_ = 86.2, *P* < 0.001). This difference was mainly due to *J. effusus*, which formed more biomass than all the other plant species.

The focal *Carex* plants responded similarly to phase 2 conditioning treatments, and so were combined in subsequent analyses. The relationship between shoot biomass in the conditioning phase and total biomass of the *Carex* phytometer in the feedback phase was positive (soil conditioned with plants typical to fen meadows: *r*^2^ = 0.034, *t*_177_ = 2.51, *P* = 0.013; soil conditioned with plants typical to degraded fen meadows: *r*^2^ = 0.333, *t*_178_ = 9.42, *P* < 0.001).

**Figure 2.**
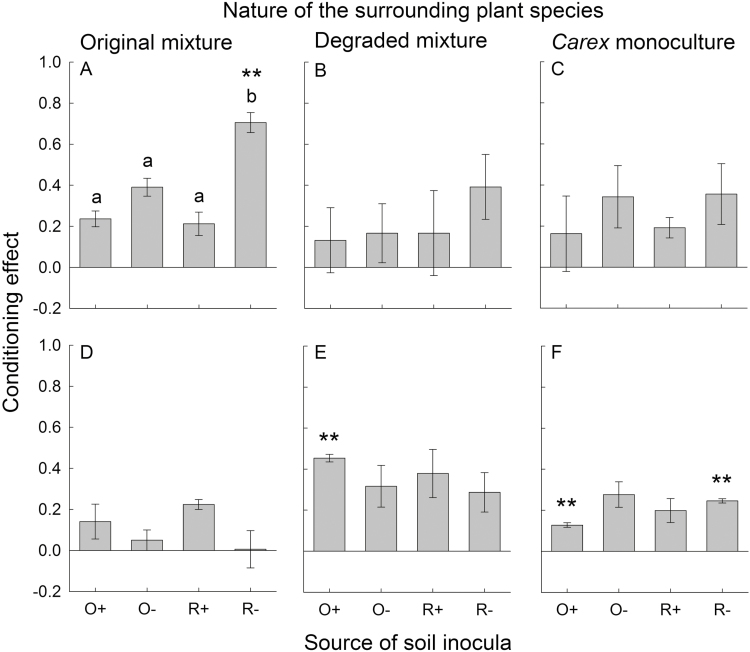
Effect of soil conditioning on *Carex* (upper row; A–C) and surrounding plant species (lower row; D–F) when grown in soil originating from different field types. The conditioning effect (mean ± SE) was calculated as ln [(total biomass when grown in soil conditioned with a mixture of plants characteristic of original fen meadows)/(total biomass when grown in soil conditioned with a mixture of plants characteristic of degraded fen meadows)]. The soil inoculum originated from four field types: original with characteristic fen meadow vegetation (O+), original, but with degraded vegetation (O−), successfully restored to characteristic fen meadow vegetation (R+) and unsuccessfully restored (R−). The plants used as surrounding neighbours were: a mixture of plants characteristic of original fen meadows (A, D), a mixture of plants characteristic of degraded fen meadows (B, E) and the same plant species (*Carex* monoculture; C, F). Asterisks indicate a significant difference from zero (*n* = 3 species averages; **P < 0.01 remaining significant after control of false discovery rate). Different letters indicate significant differences among the field types within a neighbour plant mixture.

Soil conditioning with plant species that are typical to original fen meadows compared to conditioning with plant species typical to degraded fen meadows significantly increased biomass of *Carex* phytometers, but only when neighbouring plants were also characteristic of original fen meadows and when soil originated from unsuccessfully restored fields (Fig. 2A; Tables 1 and 2). Biomass of neighbouring plants typical of original fen meadows was not responsive to soil conditioning treatment (Fig. 2D; Table 1). However, biomass of neighbouring plants typical of degraded fen meadows and biomass of *Carex* neighbours was significantly higher in soil conditioned with plant species typical of original fen meadows compared to soil conditioned with plant species typical of degraded fen meadows, although only when soil originated from original fen meadows (Fig. 2E and F; Table 1). Biomass of *Carex* neighbours was also enhanced by conditioning with species of original versus degraded meadows when soil originated from unsuccessfully restored meadows (Fig. 2F; Table 1).

**Table 1.** Results of *t*-tests analysing if effects of soil conditioning on *Carex* and surrounding plant species differed from zero (df = 2). *P*-values in bold are significant after sharpened control of false discovery rate of all tests (Benjamini and Hochberg, 2000).

Plant mixture	Soil origin	*Carex*		Surrounding	
		*t*	*P*	*t*	*P*
Original	O+	6.16	0.025	1.66	0.239
	O−	8.92	0.012	0.99	0.427
	R+	3.75	0.064	9.31	0.011
	R−	14.43	**0.0048**	0.06	0.956
Degraded	O+	0.83	0.493	24.79	**0.0016**
	O−	1.16	0.366	3.11	0.090
	R+	0.81	0.504	3.22	0.084
	R−	2.47	0.132	2.98	0.097
Monoculture	O+	0.89	0.470	11.22	**0.0079**
	O−	2.26	0.152	4.46	0.047
	R+	3.87	0.061	3.40	0.077
	R−	2.40	0.138	23.36	**0.0018**

**Table 2.** Results of ANOVA testing the effect of field type (O+, O−, R+ and R−) on the conditioning effect on *Carex* and surrounding plants.

Plant mixture	*Carex*		Surrounding plants	
	*F*_3,8_	*P*	*F*_3,8_	*P*
Original	22.92	0.0003	2.04	0.187
Degraded	0.50	0.691	0.65	0.606
Monoculture	0.50	0.695	2.21	0.162

### Germination

Germination rates of *C. hostiana* over time were overall significantly higher in soil conditioned by degraded versus original fen meadow species (*F*_19,152_ = 1.80, *P* = 0.027). However, contrary to our expectations, Tukey HSD did not reveal significant differences in germination rate at any one individual time point. Germination rates of *C. hostiana* did not significantly differ between soil from original and degraded fen meadows (inoculum from field types O+ and O−: *F*_1,8_ = 0.03, *P* = 0.86).

## Discussion

In support of our first hypothesis, our results show that conditioning soil with original fen meadow plants promotes the growth of three *Carex* spp. that are typical matrix species in fen meadows. However, plants typical of disturbed fen meadows were also promoted in soil conditioned by original fen meadow plants. Furthermore, the positive effect of soil conditioning by plants from original fen meadows on *Carex* depended on the origin of the soil: the effect occurred only when the soil originated from unsuccessfully restored fields. This is opposite to our second hypothesis, as we had assumed that soil conditioning effects would be most positive in soils from undisturbed original fen meadows. Nevertheless, this result may be of interest for restoration management, as it shows that plants that are indicative of original fen meadows may change negative soil legacy effects, which have been created by plant species growing in degraded meadows, into effects that benefit typical dominant fen meadow species. Soil conditioning effects on the phytometer *Carex* plants could have acted both directly and indirectly through effects on the surrounding plant species (Van der Putten and Peters 1997; Kardol *et al.* 2007; Petermann *et al.* 2008). However, the present experimental set-up does not allow separation of direct and indirect effects.

Original fen meadow plants are classified as later successional plant species, which usually have a neutral to positive feedback effect (Kardol *et al.* 2006; Bauer *et al.* 2015). Therefore, the lack of effect of soil conditioning on the surrounding plant species typical to original fen meadows may not be surprising. In contrast, plants typical to degraded fen meadows are classified as early successional plant species, which generally experience negative plant-soil feedback effects when grown on self-conditioned soil (Kardol *et al.* 2006). This is in agreement with the result that soil conditioning with plants typical to original fen meadows increased the biomass of neighbour plants typical to degraded fen meadows compared to conditioning with plants from degraded fields. However, it may limit possibilities for managing vegetation through influencing soil legacy effects when fen meadow plants not only promote soil conditions for themselves, but also for plants from degraded fen meadows, as effects from competition may outweigh soil legacy effects (Peltzer 2001; Sun *et al.* 2014; Larios and Suding 2015). Plants characteristic for degraded fen meadows prefer lower soil pH and higher soil fertility than the original fen meadow plants, as indicated by their Ellenberg values (*Databank Ellenbergwaarden*; accessed on 25 April 2017). Changing these abiotic soil conditions combined with influencing biotic legacy effects may hold promise as a way to decrease growing conditions and competitive ability for degraded versus original fen meadow species.

Nutrient depletion in the conditioning phase would have resulted in negative relationships between biomass in conditioning and feedback phases of the experiment. Instead, the positive relationship that we found suggests that conditioning did not influence plant performance through nutrient depletion. Incorporation of roots from the conditioning phase of the experiment in the soil of the second phase may have resulted in a transfer of compounds with a negative effect on plant performance in the feedback phase (Zhang *et al.* 2016). *Juncus effusus*, which constituted the major part of the biomass of the degraded fen meadow plants, is known to contain such so-called allelopathic compounds (Ervin and Wetzel 2003). We think that allelopathic effects of roots on *Carex* spp. are negligible in our experiment, considering the positive correlation between *J. effusus* biomass in the first phase and biomass of *Carex* spp. in the second phase of the experiment.

We were not able to trace the causes of plant biomass change down to specific soil biota. Nevertheless, our plant community-soil feedback approach suggests that adverse soil biota play a role in the feedback effect between plant species from degraded and original fen meadows. When soils are taken out of agricultural production to restore species-rich grasslands, over successional time fast-growing pathogenic fungi are replaced by slow-growing beneficial fungi (Hannula *et al.* 2017). In our experiment, conditioning with later successional plants from original fen meadows may have decreased the density of pathogenic soil fungi, while increasing beneficial fungi such as mycorrhiza. It is unclear if the phytometer *Carex* benefited from the higher mycorrhizal biomass that we found in soils conditioned with plants typical to original than to degraded fen meadows. *Carex* spp. are not mycorrhiza-dependent (Brundrett 2009), although association with mycorrhizae has been occasionally reported (Harley and Harley 1987; Muthukumar *et al.* 2004).

Our results have been obtained under controlled conditions that differ from those in the field, where water availability and other abiotic conditions can fluctuate and the presence of aboveground biota may influence the effect of soil biota on plants (Heinze *et al.* 2016). Moreover, soil collection, processing and conditioning had a substantial impact on nematodes and possibly also on other soil biota. In addition, we only found differences in mycorrhiza between the two conditioning treatments, perhaps because these changes may take more time to develop (Kulmatiski and Beard 2011). We expect that the low nematode densities in our conditioned soils had less pronounced effects on plant biomass than would have higher densities in field-collected soil. Still, our results provide new insights into why unsuccessful nature restoration may become a self-reinforcing process (Yelenik and D’Antonio 2013). Compared to plant species from original fen meadows, plant species from degraded fen meadows may create adverse biotic conditions in the soil that not only reduce the performance of themselves, but also of *Carex* species that are matrix plants in original fen meadow vegetation. Our results suggest that such negative legacy effects of soil biota from plant species that are typical of disturbed fen meadows may be altered by growing certain plant species that are typical to original fen meadows, as they can transform adverse legacy effects into positive effects. To make these results applicable for the practice of nature restoration, the challenge will be to identify plant species that can create such a change in soil biota that they enhance growth conditions of an original fen meadow plant community, whereas reducing conditions for plant species typical of disturbed fen meadows. In order to further develop such approaches, more studies on plant-soil feedback need to be carried out in the context of ecological restoration (Eviner and Hawkes 2008; Kardol and Wardle 2010).

## Conclusions

Growth conditions for *Carex* spp. that are common matrix plant species in fen meadows can be improved by growing fen meadow plant species in degraded fen meadow soils. However, plant species from degraded fen meadows may also benefit from those soil conditioning treatments. In order to make these insights applicable to fen meadow restoration, plant species need to be identified that create positive soil legacy effects to original fen meadow plant species, while creating adverse conditions for plant species from degraded fen meadows. We propose that such approach might be applied as well in the restoration of other degraded ecosystems.

## Sources of Funding

This work was funded by the former ministry of Agriculture, Nature Management and Fisheries (LNV) of the Netherlands.

## Contributions by the Authors

W.H.P., W.B. and E.P.B. conceived the idea; all authors designed the experiments; C.E.R. and E.P.B. conducted the experiments; E.P.B. analysed the results; E.P.B. and W.H.P. wrote the manuscript; and W.B. and C.E.R. commented on the manuscript.

## Conflicts of Interest

None declared.

## Supporting Information

The following additional information is available in the online version of this article—

**Appendix S1.** Analysis of soil characteristics.

**Table S1.** Geographical coordinates and soil characteristics of the fields.

**Table S2.** Plant species that were used in the feedback experiment.

**Table S3.** Abiotic and biotic soil characteristics after the conditioning phase.

**Figure S1.** PCA of nematode taxa extracted from the fields.

## Supplementary Material

Supporting Information
