# Supplementary material for: Changing soil legacies to direct restoration of plant communities
Source: AoB Plants. 2017 Aug 23;9(5):plx038. doi: 10.1093/aobpla/plx038 (PMC5597848; doi:10.1093/aobpla/plx038)
Supplement: Supporting Information [file plx038_suppl_Supporting_Information.docx]

**Appendix S1.** Analysis of soil characteristics.

**Field-collected soil**

**Abiotic soil characteristics:** A sample of 0.2 kg of field-collected soil was dried at 105 ºC to determine soil moisture. For further characterization, the soil was sieved (0.5 cm mesh size) and 0.1 kg of each soil was dried at 40 ºC. Soil pH was measured in a soil-water suspension (1:2.5 dw/v), concentration of NO_3_^-^ and NH_4_^+^ were determined in 1 M KCl-extracts (w/v 1:5), available P, K^+^ and Mg^2+^ in 0.01 CaCl_2_-extracts (w/v 1:10) and Olsen-P was measured in NaHCO_3_-extracts (Olsen & Sommers 1982). Soil organic matter (SOM) was measured as loss-on-ignition for 24 h at 430 ºC and C and N content were determined with a C-N analyzer (Thermo Flash EA 1112, Thermo Fisher Scientific Inc., Waltham, USA)**.**

**Nematodes:** The soil samples for nematode extraction were stored at 4 ºC for a maximum of two months, similar to the storage time for soil to be used in the experiment. Nematodes were extracted from 0.1 l soil by Oostenbrink elutriation (Oostenbrink 1960) followed by sieving on one 75 µm and three 45 µm sieves. The debris from the sieves was transferred to a double cotton wool filter (Hygia rapid, Hartmann AG, Heidenheim, Germany) on a sieve in a dish with a layer of tap water (Oostenbrink 1960). The nematodes were allowed to migrate through the filter for 24 h at 16-20 ºC. Nematodes in the roots of the same samples were extracted in a mist chamber for 48 h (Oostenbrink 1960). The nematodes were heat-killed and fixed in 4 % formaldehyde. Of the soil samples, 1/9 of the extracted nematodes were counted, whereas all nematodes were counted that were extracted from the roots. The nematodes were identified to family or genus level according to Bongers (1988). Nematodes were classified into trophic groups according to Yeates et al. (1993).

**Conditioned soil**

**Nematodes:** For nematode extraction, two soil cores of 1.0 cm diameter and 0-15 cm deep were taken from each pot. The soil cores from the three pots of each treatment were combined and stored at 4 ºC for one to two weeks until extraction (see extraction procedure of field-collected soil above).

**Ergosterol:** The remaining soil of the three pots was combined prior to further processing. The roots were gently removed from the soil and the attached root zone soil was shaken off and homogenized. Ergosterol was used as an indicator of fungal biomass other than arbuscular mycorrhizal fungi (AMF) in the soil (Olsson *et al.* 2003). A sample of about 1 g root zone soil was mixed with 4 ml 10 % (m/v) KOH/MeOH and stored at -20 ºC. Ergosterol was extracted with an alkaline-extraction method and measured on a Dionex HPLC equipped with a C18 reverse-phase column and a UV-detector set at 282 nm (De Ridder-Duine *et al.* 2006).

**NLFA:** As a measure of AMF, neutral lipid fatty acids (NLFA) were extracted (Boschker 2004). A mixture was used of 15 g root zone soil, which was shaken off the roots, and root plane soil, which was washed off a sample of the roots. The root sample was similar in proportion to the total root biomass as the root zone soil sample in proportion to total root zone soil. The root plane soil of the different plant species was washed in 35 ml demineralised water and transferred to a tube. The tube was centrifuged at 4800 rpm for 20 min, after which the water was removed with a pipette. The mixed root zone and root plane soil was frozen at -20 ºC, then freeze dried and stored at -20 ºC until further analysis. NLFA were extracted following the procedure described by Boschker (2004). First, total lipids were extracted from 3 g soil with a mixture of chloroform, methanol and MilliQ water (5:10:4 v/v/v). The lipids were fractionated on silicic acid columns with chloroform, acetone and methanol (1:1:2 v/v/v), dissolving neutral lipids in the chloroform fraction. With mild-alkaline derivation, fatty acid methyl esters (FAMEs) were released and analyzed by a gas-chromatography-flame ionization detector (GC_FID) on a Thermo Scientific Focus GC with a Zebron ZB5 (60 m, 0.32 mm, 0.25 µm) column. Peak areas were calculated relative to the internal standards 12:0 and 19:0. The signature fatty acid 16:1ω5 was used as marker for AMF (Olsson 1999).

For analysis of soil moisture, NO_3_^-^, NH_4_^+^ and Olsen-P, soil samples from the forty conditioned soils were stored at 4 ºC for less than a week (see extraction procedure of field-collected soil).

**Data analysis**

The nematode community in the soil collected from the field prior to phase 1 conditioning was analysed with principal component analysis (PCA), using the CANOCO programme version 4.55 (ter Braak and Šmilauer 2006). Numbers of nematodes were ln(x+1)-transformed before analysis, as this gives less weight to dominant species and therewith concentrates on qualitative aspects (Jongman *et al.* 1995). Differences in abiotic characteristics of field-collected soil among the four types of fen meadows were analyzed by one-way ANOVA using Statistica 10 (Statsoft Inc.). At the end of the conditioning phase, differences in effects of the conditioning plant mixtures on nematode trophic groups (Yeates *et al.* 1993), ergosterol and NLFA content as well as Olsen-P and NH_4_+NO_3_ in the soil were analyzed by t-tests in Statistica. False Discovery Rates were controlled with the sharpened method of Benjamini and Hochberg (2000). This method reduces the chance of making type I errors, while having more power than classical Bonferroni-type control of family-wise error rate (Verhoeven *et al.*, 2005).

**REFERENCES**

Benjamini Y, Hochberg Y. 2000. On the adaptive control of the false discovery rate in multiple testing with independent statistics. *Journal of Educational and Behavioral Statistics* **25**: 60-83.

Bongers T. 1988. *De nematoden van Nederland*. Schoorl, The Netherlands: Pirola.

Boschker, H.T.S. 2004. Linking microbial community structure and functioning: stable isotope (13C) labeling in combination with PLFA analysis. *Molecular microbial ecology manual* (eds G.A. Kowalchuk, F.J. De Bruijn, I.M. Head, A.D. Akkermans & J.D. van Elsas). Kluwer academic publishers, Dordrecht, The Netherlands.

De Ridder-Duine, A.S., Smant, W., Van der Wal, A., Van Veen, J.A. & De Boer, W. 2006. Evaluation of a simple, non-alkaline extraction protocol to quantify soil ergosterol. *Pedobiologia* **50**: 293-300.

Jongman RHG, ter Braak CJF, Van Tongeren OFR. 1995. *Data analysis in community and landscape ecology.* Cambridge: Cambridge University Press.

Olsen, S.R. & Sommers, L.E. 1982. Phosphorus. *Methods of soil analysis (part 2)* (eds A.L. Page, R.H. Miller & D.R. Keeney), pp. 403-430. ASA-SSSA, Madison, WI, USA.

Olsson, P.A. 1999. Signature fatty acids provide tools for determination of the distribution and interactions of mycorrhizal fungi in soil. *Fems Microbiology Ecology* **29**: 303-310.

Olsson, P.A., Larsson, L., Bago, B., Wallander, H. & van Aarle, I.M. 2003. Ergosterol and fatty acids for biomass estimation of mycorrhizal fungi. *New Phytologist* **159**: 7-10.

Oostenbrink, M. 1960. Estimating nematode populations by some selected methods. *Nematology* (eds J.N. Sasser & W.R. Jenkins), pp. 85-102. The University of North Carolina Press, Chapel Hill, USA.

ter Braak CJF, Šmilauer P. 2006. *Canoco for windows version 4.55.* Wageningen, The Netherlands: Biometris, WUR.

Verhoeven KJF, Simonsen KL, McIntyre LM. 2005. Implementing false discovery rate control: increasing your power. *Oikos* **108**: 643-647.

Yeates, G.W., Bongers, T., De Goede, R.G.M., Freckman, D.W. & Georgieva, S.S. 1993. Feeding habits in soil nematode families and genera - an outline for soil ecologists. *Journal of Nematology* **25**: 315-331.

Table S1. Geographical coordinates (decimal) and soil characteristics (0-20 cm) of the fields at the time of sampling January-February 2008. Lat. = latitude (°N), Long. = longitude (°E). Soil was sampled from five fields each of four field types: original with typical fen meadow vegetation (O+), original, but with degraded vegetation (O-), successfully restored to typical fen meadow vegetation (R+), unsuccessfully restored (R-). There were no significant differences among the soil types.

|  | Lat. | Long. | pH H_2_O | %  moisture | %  Organic matter | % C | % N | mineral N  mg ∙ kg^-1^ | %NO_3_-N | P | Olsen- P | K | Mg |
| --- | --- | --- | --- | --- | --- | --- | --- | --- | --- | --- | --- | --- | --- |
|  |  |  |  |  |  |  |  |  |  | (mg ∙ kg^-1^) | | | |
| Background soil | 52.11 | 6.45 | 5.5 | 20 | 1.4 | 0.9 | 0 | 1.0 | 0 | 0.03 | 7.4 | 7.5 | 15 |
| O+ |  |  |  |  |  |  |  |  |  |  |  |  |  |
| Groot Zandbrink | 52.13 | 5.48 | 5.5 | 68 | 5.8 | 3.3 | 0.19 | 20.1 | 0 | 0.28 | 2.5 | 20.4 | 97 |
| Lemselermaten | 52.35 | 6.88 | 6.2 | 140 | 24.8 | 13.4 | 0.97 | 21.1 | 12 | 0.34 | 7.6 | 38.9 | 126 |
| De Marschen | 53.06 | 6.17 | 4.9 | 92 | 16.7 | 10.3 | 0.59 | 55.5 | 0 | 0.12 | 3.9 | 35.7 | 25 |
| Allemanskampje | 52.06 | 5.56 | 7.7 | 82 | 9.6 | 5.5 | 0.35 | 10.5 | 45 | 0.01 | 3.8 | 11.3 | 36 |
| Stelkampsveld | 52.12 | 6.48 | 6.4 | 82 | 8.5 | 5.6 | 0.28 | 14.6 | 0 | 0.01 | 3.6 | 21.3 | 54 |
| O- |  |  |  |  |  |  |  |  |  |  |  |  |  |
| Groot Zandbrink | 52.13 | 5.48 | 4.6 | 63 | 4.6 | 2.9 | 0.14 | 15.5 | 0 | 0.40 | 5.1 | 22.4 | 23 |
| Lemselermaten | 52.35 | 6.88 | 6.4 | 204 | 16.1 | 9.1 | 0.65 | 22.3 | 29 | 0.28 | 6.7 | 37.2 | 142 |
| Allemanskampje | 52.06 | 5.57 | 4.7 | 53 | 6.1 | 3.7 | 0.20 | 13.6 | 0 | 0.07 | 2.6 | 29.4 | 19 |
| Zwartebroek | 52.18 | 5.52 | 5.1 | 51 | 5.6 | 3.1 | 0.18 | 4.1 | 12 | 0.09 | 3.3 | 17.9 | 83 |
| Koolmansdijk | 52.02 | 5.57 | 5.0 | 38 | 4.4 | 3.0 | 0.22 | 15.7 | 0 | 0.01 | 2.5 | 15.4 | 11 |
| R+ |  |  |  |  |  |  |  |  |  |  |  |  |  |
| Meeuwenkampje | 52.05 | 5.55 | 5.1 | 60 | 11.6 | 7.0 | 0.34 | 18.2 | 19 | 0.27 | 16.8 | 24.9 | 31 |
| Punthuizen | 52.35 | 7.05 | 5.8 | 60 | 5.6 | 3.2 | 0.17 | 26.0 | 0 | 0.13 | 4.0 | 27.7 | 20 |
| Lemselermaten | 52.35 | 6.88 | 6.4 | 39 | 2.9 | 1.4 | 0.06 | 3.4 | 2 | 0.00 | 1.6 | 28.7 | 79 |
| Stroothuizen | 52.37 | 7.05 | 4.9 | 82 | 9.0 | 4.1 | 0.24 | 21.8 | 0 | 0.22 | 53.3 | 43.9 | 72 |
| Wijnjeterperschar | 53.06 | 6.17 | 5.7 | 108 | 13.9 | 9.6 | 0.54 | 70.5 | 1 | 0.00 | 4.6 | 30.2 | 64 |
| R- |  |  |  |  |  |  |  |  |  |  |  |  |  |
| Veenkampen | 51.98 | 5.62 | 5.6 | 118 | 20.4 | 8.5 | 0.74 | 24.9 | 23 | 0.66 | 8.7 | 28.2 | 165 |
| Lemselermaten | 52.35 | 6.87 | 6.6 | 67 | 5.2 | 2.8 | 0.15 | 1.8 | 15 | 0.00 | 2.6 | 17.9 | 81 |
| Stroothuizen | 52.37 | 7.05 | 4.2 | 49 | 5.4 | 3.9 | 0.13 | 1.6 | 4 | 0.00 | 1.3 | 16.9 | 17 |
| De Marschen | 53.06 | 6.17 | 4.3 | 95 | 16.9 | 11.4 | 0.65 | 38.0 | 9 | 0.24 | 7.2 | 41.2 | 34 |
| Loefvledder | 53.08 | 6.67 | 4.2 | 85 | 21.0 | 16.7 | 0.60 | 30.6 | 11 | 0.16 | 5.4 | 46.5 | 30 |

Table S2. Plant species that were used in the feedback experiment with an indication of functional group, specificity to fen meadows and Ellenberg values of moisture (F), soil pH (R) and soil fertility (N). Plants typical to original and to degraded fen meadows were used to condition the soil in phase 1 and as surrounding plants in phase 2, whereas the focal plants were used as phytometers in phase 2 of the experiment.

| Experimental group | |  | Specificity to fen meadows^1^ | Ellenberg values^2^ | | | Experimental phase^3^ | |
| --- | --- | --- | --- | --- | --- | --- | --- | --- |
|  | Plant species | Group |  | F | R | N | 1 | 2 |
| Focal plants (phase 2) | |  |  |  |  |  |  |  |
|  | *Carex flacca* | Sedge |  | 6 | 8 | 4 | 0 | 1 or 4 |
|  | *Carex panicea* | Sedge | kA | 9 | 6 | 2 | 0 | 1 or 4 |
|  | *Carex hostiana* | Sedge | kA | 8 | x | 4 | 0 | 1 or 4 |
| Plants typical to original fen meadows | |  |  |  |  |  |  |  |
|  | *Festuca rubra* ssp. *commutata* | C3 grass | dK | 6 | 6 | x | 3 | 1 |
|  | *Juncus conglomeratus* | Rush | kO | 7 | 4 | 3 | 1 | 0 |
|  | *Luzula multiflora* | Rush | kO | 5 | 5 | 3 | 9 | 1 |
|  | *Succisa pratensis* | Forb | kV | 7 | x | 2 | 9 | 1 |
| Plants typical to degraded fen meadows | |  |  |  |  |  |  |  |
|  | *Filipendula ulmaria* | Forb | dO | 8 | x | 5 | 2 | 1 |
|  | *Juncus effusus* | Rush |  | 7 | 3 | 4 | 8 | 1 |
|  | *Lysimachia vulgaris* | Forb |  | 8 | x | x | 1 | 1 |

^1^ Classification of specificity to fen meadows (association of *Cirsio dissecti-Molinietum*) of the plant species following Schaminée et al. 1996: k (character-taxon that is more common in one vegetation type than in others in an area); d (differential taxon that distinguishes one vegetation type from another); level of classification of the vegetation types: K (class); O (order); V (alliance); A (association).

^2^ Ellenberg values from *Databank Ellenbergwaarden* at www.biw.kuleuven.be (accessed on 25-4-2017). F (moisture; scale 1-12), R (soil pH; scale 1-9), N (nitrogen; scale 1-9), x=broad amplitude.

^3^ Number of plants in the three conditioning pots together in phase 1, or per pot in phase 2 of the experiment. In phase 2, the focal plants were surrounded by a mixture of plants typical to original or degraded fen meadows, or three plants of the same species (*Carex* monoculture).

Table S3. Average and 95% confidence interval of abiotic and biotic soil characteristics after soil conditioning with plant species that are typical to original or to degraded fen meadows, as well as t- and P-values of t-tests (n=20) of pairwise differences in effect on the soils. Abiotic soil characteristics and ergosterol are presented in mg ∙ kg^-1^, NLFA in nmol ∙ g^-1^, and nematodes in numbers ∙ kg^-1^. P-values in bold are significant after control of False Discovery rate.

|  | Conditioning plants | |  |  |
| --- | --- | --- | --- | --- |
| Soil characteristic | Original | Degraded | t | P |
| NH_4_ + NO_3_ | 9.9 (7.0-12.8) | 9.5 (7.0-12.0) | 0.70 | 0.494 |
| Olsen-P | 7.2 (4.2-10.2) | 6.9 (3.8-9.9) | 1.70 | 0.106 |
| Ergosterol | 0.41 (0.26-0.56) | 0.39 (0.26-0.52) | 0.49 | 0.633 |
| NLFA | 12.4 (8.5-16.3) | 5.1 (1.1-9.1) | 3.84 | **0.001** |
| Total nematodes | 580 (441-720) | 463 (324-603) | 2.12 | 0.048 |
| Endoparasites | 44 (9-78) | 20 (4-36) | 1.35 | 0.191 |
| Ectoparasites | 17 (9-25) | 36 (6-65) | -1.64 | 0.117 |
| Root hair feeders | 142 (78-206) | 102 (59-144) | 2.22 | 0.039 |
| Bacterial feeders | 245 (171-319) | 194 (138-250) | 1.42 | 0.171 |
| Fungal feeders | 92 (51-134) | 90 (48-132) | 0.10 | 0.918 |
| Omnivores/  predators | 41 (21-61) | 22 (13-30) | 2.72 | 0.014 |

**Figure S1.** PCA of nematode taxa extracted from soil from four field types: original with typical fen meadow vegetation (O+; fields 1-5), original, but with degraded vegetation (O-; fields 6-10), successfully restored to typical fen meadow vegetation (R+; fields 11-15), unsuccessfully restored (R-; fields 16-20). The explained variation of the x-axis is 19.4 % and of the y-axis is 14.2 %.

Angui = Anguinidae, Aphe = Aphelenchidae, Aphe_oi = Aphelenchoididae, Araeo = Araeolaimina, Ceph_dae = Cephalobidae, Ceph_ina = Cephalobina (other than Cephalobidae), Crico = Criconematidae, Diphther = Diphtherophoridae, Diplogas = Diplogasterina, Dolicho = Dolichodoridae, Dorylai = Dorylaimina, Ecphya = Ecphyadophoridae, Hemi = Hemicycliophoridae, Hetero = Heteroderidae, Hoplo = Hoplolaimidae, Monhys = Monhysterina, Monon = Mononchina,Plect = Plectidae, Prismato = Prismatolaimidae, Para = Paratylenchidae, Prat = Pratylenchidae, Rhab_ina = Rhabditina, Terato = Teratocephalidae, Tricho = Trichodoridae, Tripyl = Tripylina,Tyl_idae = Tylenchidae, Tyl_ina = Tylenchina (other).
